# The complete chloroplast genome of *Echeveria lilacina* Kimnach & Moran 1980 (Saxifragales: Crassulaceae)

**DOI:** 10.1080/23802359.2022.2077668

**Published:** 2022-05-26

**Authors:** Gyoungju Nah, Ji Ran Jeong, Jae Hwan Lee, Soon Yil Soh, Sang Yong Nam

**Affiliations:** a Genome Analysis Center at National Instrumentation Center for Environmental Management, Seoul National University, Seoul, Korea; b Department of Environmental Horticulture, Sahmyook University, Seoul, Korea; c Natural Science Research Institute, Sahmyook University, Seoul, Korea

**Keywords:** *Echeveria lilacina*, Crassulaceae, complete chloroplast genome, next generation sequencing, phylogenetic analysis

## Abstract

*Echeveria lilacina* Kimnach & Moran 1980 is a succulent plant having ornamental and ecological importance. In this study, the first complete chloroplast genome of *Echeveria lilacina,* a species belonging to the Crassulaceae family, was characterized from the *de novo* assembly of Illumina NovaSeq 6000 paired-end sequencing data. The chloroplast genome of *E. lilacina* is 150,080 bp in length, which includes a large single-copy (LSC) region of 81,741 bp, a small single-copy (SSC) region of 16,747 bp, and a pair of identical inverted repeat regions (IRs) of 25,796 bp each. The genome annotation revealed a total of 138 genes, including 87 protein-coding genes, 41 transfer RNA (tRNA) genes, and 10 ribosomal RNA (rRNA) genes. The phylogenetic analysis with 15 complete chloroplast genome sequences including outgroup showed that *E. lilacina* formed the closest taxonomical relationship with *Graptopetalum amethystinum* in the Crassulaceae family.

*Echeveria*, a member of the Crassulaceae family, is a genus consisting of approximately 150 succulent species. Of them, *Echeveria lilacina* Kimnach & Moran 1980 (Kimnach and Moran 1980), also called Ghost Echeveria, has been often used as an ornamental plant and is known for an environmental host plant for butterflies (Ziegler and Escalante 1964). *Echeveria lilacina* is characterized by pale and silvery-gray fleshy leaves and pale pink or coral-pink flowers from late winter to early spring (López-Angulo et al. 2022). The habitat of *E. lilacina* ranges from semi-desert areas of west Texas to Argentina, across Mexico, Guatemala, and Central and South America (Palomino et al. 2021).

The sample of *E. lilacina* plant was collected from Nuevo León, Mexico (25°06′50.0″N, 100°11′50.0″W) in accordance with local regulation and permission of local authorities. The collected plant was deposited at National Agrobiodiversity Center at Rural Development Administration (http://genebank.rda.go.kr/distribGuide.do, Mun Sup Yoon, msyoon63@korea.kr), Korea under the genetic resource number IT317443. The specimen of *E. lilacina* under the voucher number SYCS101 was deposited at Natural Science Research Institute (https://www.syu.ac.kr/industry-academic-research/a-research-institute/natural-science-institute/#, contact person: Jae Hwan Lee, dlwoghks1236@naver.com), Sahmyook University, which is the affiliated organization of National Agrobiodiversity Center at Rural Development Administration, Korea and serves as the institute of agricultural genetic resources for succulent plants.

The leaves of *E. lilacina* were used to extract genomic DNA using a modified CTAB-based protocol, followed by DNA QC by Nanodrop and agarose gel electrophoresis. The genomic library for Illumina 150 bp paired-end (PE) was constructed according to the manufacturer’s recommendation using NEXTflex^®^ Rapid DNA sequencing kit (Bioo Scientific, Austin, TX, USA). The chloroplast genome of *E. lilacina* was sequenced by NovaSeq 6000 (Illumina Inc., San Diego, CA, USA) and low-quality and adaptor sequences were removed by Trimmomatic (Bolger et al. 2014). The high-quality PE reads were assembled by CLC Genomics Workbench (ver. 10.0.1, CLC QIAGEN), followed by manual curation through PE reads mapping (Kim et al. 2015). Annotation of the complete chloroplast genome was performed with GeSeq and manual corrections (Tillich et al. 2017). The complete chloroplast genome sequence of *E. lilacina* was submitted to GenBank with the accession number MZ643065.

The complete chloroplast genome of *E. lilacina* was 150,080 bp in length with 37.88% of G + C content, comprising a large single copy (LSC) region of 81,741 bp, a small single copy (SSC) region of 16,747 bp, and a pair of inverted repeats (IRa and IRb) regions of 25,796 bp each. The genome contained 138 genes including 87 protein-coding genes, 41 tRNA genes, and 10 rRNA genes.

In order to investigate the evolutionary relationship, the complete chloroplast genome sequences of *E. lilacina* with 14 related species in Crassulaceae and one outgroup species, were aligned using ClustalW (ver. 2.1) (Larkin et al. 2007), followed by phylogenetic tree construction based on Maximum Likelihood (ML) algorithm with 1000 bootstraps using MEGA 10.2.5 (Kumar et al. 2018). The phylogenetic analysis showed the closest relationship of *E. lilacina* with *Graptopetalum amethystinum* in the family of Crassulaceae (Figure 1).

**Figure 1.**
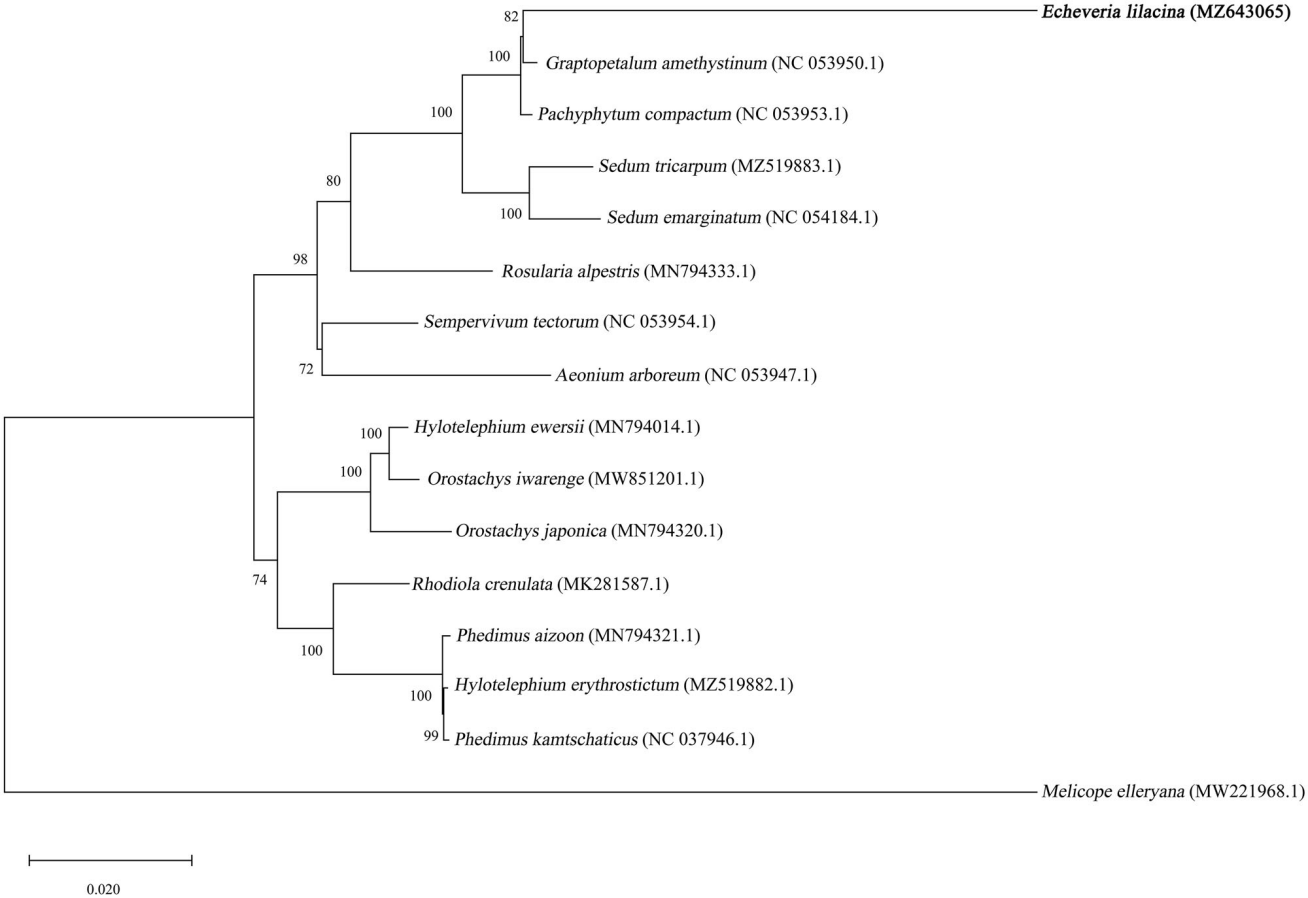
Maximum-likelihood phylogenetic tree based on complete chloroplast genome sequences of *E. lilacina* (bold font) and 14 related species with one outgroup species, *Melicope elleryana*. The GenBank accession numbers are designated next to each species name. The values above branches are bootstrap percentages based on 1000 replicates.

## Author contribution

SYN conceived the original structure of the project and reviewed the manuscript. JHL prepared the plant sample and SYS prepared the DNA of the sample. JRJ analyzed the data. GN interprets the data and prepared the manuscript. All authors have read and agreed to the published version of the manuscript.

## Data Availability

The data that support the finding of this study are publically available in GenBank at http://www.ncbi.nlm.gov/genbank/, with reference number, MZ643065. The BioProject, BioSample, and SRA numbers are PRJNA751436, SAMN20518232, and SRR15321362, respectively.
